# Variant Tool Chest: an improved tool to analyze and manipulate variant call format (VCF) files

**DOI:** 10.1186/1471-2105-15-S7-S12

**Published:** 2014-05-28

**Authors:** Mark TW Ebbert, Mark E Wadsworth, Kevin L Boehme, Kaitlyn L Hoyt, Aaron R Sharp, Brendan D O'Fallon, John SK Kauwe, Perry G Ridge

**Affiliations:** 1 Department of Biology, Brigham Young University, Provo, Utah, USA; 2 ARUP Institute for Clinical and Experimental Pathology, Salt Lake City, Utah, USA

## Abstract

**Background:**

Since the advent of next-generation sequencing many previously untestable hypotheses have been realized. Next-generation sequencing has been used for a wide range of studies in diverse fields such as population and medical genetics, phylogenetics, microbiology, and others. However, this novel technology has created unanticipated challenges such as the large numbers of genetic variants. Each caucasian genome has more than four million single nucleotide variants, insertions and deletions, copy number variants, and structural variants. Several formats have been suggested for storing these variants; however, the variant call format (VCF) has become the community standard.

**Results:**

We developed new software called the Variant Tool Chest (VTC) to provide much needed tools to work with VCF files. VTC provides a variety of tools for manipulating, comparing, and analyzing VCF files beyond the functionality of existing tools. In addition, VTC was written to be easily extended with new tools.

**Conclusions:**

Variant Tool Chest brings new and important functionality that complements and integrates well with existing software. VTC is available at https://github.com/mebbert/VariantToolChest

## Background

The variant call format (VCF) has become the standard format for storing variants identified in next-generation sequencing (NGS) and other studies. VCF files are flexible with eight fixed fields including chromosome (CHROM), position (POS), known variant IDs such as dbSNP identifications (ID), reference allele (REF), alternate allele(s) (ALT), variant quality score (QUAL), filter information summarizing why a variant was or was not considered valid by the variant calling software (FILTER), and an information field (INFO). Additional fields containing genotypes for one or more samples may also be present. Each row of the file contains information about observed variants at the given position and chromosome, may have information about how the variant(s) was/were identified (allele frequency, depth, strand bias, genotype likelihoods, etc.), and biological annotations (gene, variant frequency, 1000 Genomes membership, mRNA and protein positions, etc.). The last columns of a VCF file contain genotype information specifying whether the individual is heterozygous, homozygous reference or variant, or whether it is unknown (missing). Finally, VCF files can contain information for a single or multiple samples. Alternatively, summary VCF files containing minimal information (chromosome, position, reference allele, variant allele, and genotypes) can be used. VCF files are used to store all variant types including single nucleotide variants, insertions and deletions, copy number variants, and structural variants. The VCF has become an important format in modern biology and is the only widely used format for variant storage.

Several programs exist for manipulating and comparing VCF files: VCF tools [1], BedTools [2], BcfTools, and the Genome Analysis Toolkit (GATK) [3,4]. Each of these softwares is flexible and powerful, but missing certain essential features. In this manuscript we describe a novel program, the Variant Tool Chest (VTC). The Variant Tool Chest complements existing softwares by extending their capabilities without replicating existing solutions for working with VCF files. We also provide suggestions for building upon the VTC rather than building new tools from scratch. VTC can be downloaded at https://github.com/mebbert/VariantToolChest.

## Results and discussion

### Novel features

#### Multi-sample VCF support

As next-generation sequencing continues to gain momentum, researchers need the ability to compile many samples into a single VCF or analyze variants from multiple VCF files. VTC was built to work with a combination of multi- and single-sample VCF files. Existing softwares are only capable of handling either a single VCF file, or one multi-sample VCF file. VTC can handle a mix of single and multi-sample VCF files, with the user defining which sample(s) to use from each of the VCF files.

#### Genotype set operations

VTC contains a powerful set operation tool named "SetOperator" designed to perform simple or complex set operations using VCF files, including intersects, complements, and unions. While various tools exist to perform set operations on VCF files, VTC improves existing solutions in two ways. First, existing software performs set operations based only chromosome and base pair position. This means that if one individual is heterozygous and another homozygous, the resulting operations would assume that these two individuals have the same genotype. Second, existing tools work on only a collection of single sample VCF files. In contrast, VTC can perform set operations on a single multi-sample VCF file, or a combination of multi- and single sample VCF files. Furthermore, the user can choose to only perform the operations based on certain individuals from each multi-sample VCF file. These abilities save researchers time by not forcing the user to extract all samples of interest into a collection of single sample VCF files, and allow more efficient storage of genotypes in multi-sample VCF files. For example, it is helpful and makes sense for a researcher to store all genotypes for a single family in a single VCF file; however, the researcher may have interest in performing set operations across multiple families (VCF files), such as performing an intersection of variants from all affected individuals from all families.

VTC has several operation-specific settings for intersects and complements that allow researchers to specify genotype-level requirements. For intersects, VTC currently has five genotype-level intersect methods and two record-level (i.e., ignore genotypes) intersect methods. The genotype-level intersect methods are as follows: (1) heterozygous; (2) homozygous variant; (3) heterozygous or homozygous variant; (4) homozygous reference; and (5) match sample exactly across variant pools. The record-level intersect methods are: (1) variant; and (2) position.

The genotype-level intersect methods require that all sample genotypes involved in the intersect fall into the specified category. One caveat is that the heterozygous genotype requires the sample to have a reference allele. So if a sample's genotype has two different variant alleles (i.e. a tri-allelic position), though technically a heterozygote, will not be considered as such. This distinction is made assuming that researchers interested in identifying heterozygotes will assume the samples have a reference allele. This also greatly simplifies several corner cases when dealing with multiple variants at a single location.

The record-level intersect methods ignore genotypes and only consider whether the variant pools included in the analysis contain the variant. The "position" method only considers chromosome, position, and the reference allele, while the "variant" method also includes the alternate allele(s). For the "variant" method, records with multiple alternates are considered to intersect if at least one of the alternates matches.

There are currently three complement methods: (1) heterozygous or homozygous variant; (2) exact genotype matches; and (3) variant. When performing a complement, the "heterozygous or homozygous variant" method requires that all sample genotypes in both variant pool be either a heterozygous or homozygous variant in order to be removed from the variant pool being subtracted from. The "exact genotype" method requires that all samples across both variant pools have the same genotype, whatever it may be. The "variant" method ignores genotypes and only subtracts if the chromosome, position, reference, and alternate match between the two variant pools.

Unions combine all variants and specified samples into a single VCF file regardless of genotype. Samples missing variants will have a "no call" genotype ("./.").

#### Detailed set operation syntax

The Set Operator tool in the VTC empowers researchers to define set operations with a powerful, simple syntax. This simple syntax has several advantages: (1) researchers may specify any number of input files (variant pools) to perform operations; (2) researchers may specify specific samples within a given variant pool to include in the operation; and (3) each operation is assigned an identification value (ID) automatically by VTC or specified by the user, so that it can be used in subsequent operations. The general syntax structure for a single operation is as follows (no spaces):

oId=operator[input_id1[sample_id1,sample_id2,etc.]:input_id2[sample_id3,sample_id4,etc.]:etc.]

Where oId is a user-specified ID for the operation (may be omitted), operator is the operation of interest (i, c, or u for intersect, complement, or union), input_id is the variant pool ID, and sample_id is a sample ID for a sample within the given variant pool. If sample IDs are omitted, Set Operator will use all samples within the variant pool. For example, the following intersect operation will perform an intersect on all samples within the variant pools named "file1" and "file2": myOP=i[file1:file2].

#### Operation stringing

As previously mentioned, the set operation syntax allows resulting variant pools to be used in subsequent operations. This feature allows researchers to obtain final results with a single command in most circumstances. Continuing with the previous example, "myOP" may be specified in a subsequent operation as follows: "myOP=i[file1:file2] myOP2=c[myOP:file3]".

#### Intermediate files

When performing complex set operations, researchers may want all intermediate operation results to be printed to a file. Otherwise, the researcher would be required to perform separate commands. As such, a simple option named "--intermediate-files" will print each operation result to a file named according to the specified "oId" previously mentioned.

#### Header repair

VCF files can be complex, and maintaining a valid VCF header can be challenging. Since VTC is built on the code that defines VCFs, it is possible to detect invalid VCF headers and repair them. VTC will automatically add missing required header information such as the "GT" header line when genotypes are being printed. There are many useful (unrequired) header lines that cannot be anticipated, however. This feature is still under active development.

#### Add/remove "chr"

Chromosome numbers in VCF files may be prefixed by "chr" or may simply be the chromosome ID (e.g., chrX or X). Many next-generation sequencing softwares are incapable of handling VCF files that do not use the same convention simultaneously. For example, if one file includes "chr" and another does not, current tools will reject the files. And some tools require the VCF files to have the same chromosome ID as the reference sequenced used in the original analysis. VTC will either prepend or remove "chr" from all variant records seamlessly according to the user's specifications by simply including (or omitting) the "--add-chr" flag.

#### Summary information

Several tools exist that will provide high- or low-level detail on a variant pool, but they can be cumbersome. VTC has a tool named VarStats that will provide a quick summary of the variant pool, or a detailed variant-by-variant summary. High-level summary metrics include total number of variants, total number of single nucleotide variants (SNVs), insertions and deletions, structural variants, and variants with multiple alternates. The summary also includes summary depth and quality values. The variant-by-variant summary includes allelic counts and the minimum, maximum, and average read depth and quality scores for each variant.

#### Compare operation

Many analyses require researchers to perform several set operations to identify all variants in common between VCFs, those that are unique to a given VCF, and researchers may also need the combined set. Researchers are generally not satisfied knowing only the number of variants that fall into each group, such as would be represented by a Venn diagram. To obtain all of this information a researcher would perform four set operations: an intersect (common variants), two complements (unique variants), and a union (combined set). Set Operator has a compare operation ("--compare") that will automatically perform all four operations, print the results to their respective files, and print a summary of each resulting variant pool to the console. This option currently is limited to two input files.

#### VCF association analysis

Association analyses are common using genomic data, but we are not aware of any available tools to perform such analyses on VCF files. The VarStats tool in VTC will perform association analyses on all variants in a variant pool if a phenotype file is provided. Results, including odds ratios and p-values for each variant are printed to a file. If there are multiple alternates at a given location, VarStats will perform the analysis on each alternate and print results on a separate line. This option does not currently provide p-value correction such as multiple test correction, but will be implemented in a future release. These corrections can be easily performed in statistical software.

### Future directions

#### Filter tool

Next-generation sequencing variants are often filtered on various values including quality scores and depth. Several tools already exist that, when combined, satisfy most needs for filtering variants. Ideally, a single tool would incorporate all of this functionality along with new features for simplicity.

#### File formats

While VCFs are the most common format for next-generation sequencing variants, there are other file formats that will be incorporated into VTC including Plink (ped/map or bim/bam/fam) and comma-separated value (CSV) files. Plink is particularly important since there are many existing large-datasets in Plink format. In order to compare or combine data in Plink format to those in VCF format, there must be a tool to handle this. VTC will enable researchers to read in variant data from multiple formats and perform all of the same analyses seamlessly. This is especially pertinent as a common QC measure of single nucleotide variants identified in NGS studies is to compare NGS variants to variants genotyped on a SNP array. Array data is most often reported in Plink format.

#### Enhanced compare

As different technologies are compared, there is a need to determine concordance between samples tested on multiple technologies. VTC will implement an "Enhanced Compare" option that will report genotypes that are perfect matches, imperfect matches (heterozygous variant observed on one technology and homozygous variant observed from the other), and no matches for the same samples on different technologies.

#### Additional SetOperator options

Anticipating all possible uses and hypotheses is difficult with any new tool, especially with data as complex as genomic variants. Responding to these needs is important and will likely involve updated SetOperator options. A few options we plan to implement are to accommodate specialized union operations, similar to those for intersect and complement. Specifically, users may need to union only heterozygotes, heterozygotes or homozygous variant, only homozygous variant, or only homozygous reference.

#### Incorporate new and existing tools

Building useful computational tools that interface well together benefits researchers across all disciplines. New tools, while generally valuable to the research community, often do not integrate well with other tools used within a discipline, causing end users grief. There are likely many reasons for this fragmentation, but we would like to address two major sources: (1) contributing to an existing project can be costly (in time and money) and difficult; and (2) computational researchers need to publish their work to demonstrate academic productivity.

While object-oriented programming mitigates much of the difficulty, contributing to an existing project is still difficult because of the time and effort required to become familiar with existing source code. Many projects have hundreds of classes with complex interactions that make adding new functionality daunting. In many cases, a researcher may opt to write a separate tool simply because it is more feasible. Unfortunately, this causes fragmentation between tools. To promote well-integrated tools, VTC was written specifically to facilitate contribution with its easily extensible code structure. Any computational researcher can begin a new tool without needing to familiarize him/herself with other complex code.

Contributing to existing source code can be challenging, but publishing requirements also present a challenge to computational researchers, since publications are an essential measure of academic productivity. If a computational researcher adds a novel algorithm to an existing tool, s/he may forfeit the opportunity to publish the algorithm and get feedback from the community. Because VTC is simply a collection of useful tools, however, researchers can contribute an independent tool or algorithm with an independent name and publish it independently.

As we mentioned above, it is not possible to predict all possible operations and uses for software like VTC and we anticipate the need for additional functionality. To this end, we invite all computational researchers to contribute independent tools associated with variant analysis to VTC. This will benefit researchers by promoting tool integration within a simple, intuitive framework.

## Conclusions

VCF files are the standard format for storing variants identified in next-generation sequencing (NGS) and other studies, but working with them can be challenging. In this manuscript we describe a novel program, the Variant Tool Chest (VTC). The Variant Tool Chest is easily extendable and complements existing softwares by extending their capabilities without replicating existing solutions for working with VCF files. VTC is available at https://github.com/mebbert/VariantToolChest

## Methods

### Variant tool chest overview

The Variant Tool Chest (VTC) is a collection of tools to analyze variants from next-generation sequencing (NGS) and other studies, and is intended to become a tool chest to accommodate most analysis needs. It is written in Java (version 1.7) for speed and portability. Two tools currently exist in the tool chest named SetOperator and VarStats. Set Operator performs set operations such as intersects, complements, and unions on variant sets termed variant pools. VarStats performs statistical operations including association analyses and summaries on variant pools. Since there are numerous other tools necessary for analyzing variant pools, VTC was written with an emphasis on extensibility.

### Extensibility

To make VTC easily extensible, each tool is written independently and is self-contained within a single Java package. Researchers can add tools without being forced to familiarize and integrate with other complicated code. A single class named VTCEngine is the main entry for all tools. VTCEngine receives user input and executes the appropriate tool(s). Most arguments are passed to, and handled by the tool of interest. Each tool uses a simple argument-parsing library named Argparse4j [5] to define and handle all arguments. All tools use the same variant and sample data structures known as VariantPool and SamplePool, respectively.

**Figure 1:**
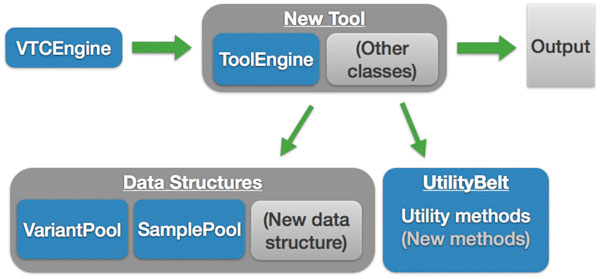
**Variant Tool Chest (VTC) was built to be extensible**. Each new tool only needs to interface with a few simple classes and is otherwise completely independent. All tools should be self-contained within a single parent Java package. The main driver class for VTC is VTCEngine. Any new tool should have its own Engine class and be instantiated from VTCEngine. All generally applicable data structures such as VariantPool and SamplePool are placed within the vtc.datastructure Java package. Any new generally applicable data structures should also be placed in vtc.datastructure. Otherwise the data structure should be housed within the tool's package. Likewise, any generally applicable methods should be placed in the UtilityBelt class.

VariantPool is built on the open source public application programming interfaces (APIs) distributed by the Broad Institute that define the Variant Call Format (VCF) file structure. Specifically, the VTC is built on the Picard [6], SAMTools [7], tribble, and variant APIs. Tribble provides necessary utilities for creating and working with various data file types, including VCF indexes. All three libraries are essential components incorporated into the Genome Analysis Toolkit (GATK) [3,4]. As such, VTC is capable of reading and writing valid VCF files, dependably. For generalizability, data structure classes are contained within the main vtc.datastructures Java package. Any future classes generally applicable across multiple tools should also be defined within the vtc.datastructures package. Likewise, a class named UtilityBelt was created for methods that are generally applicable. The file structure of VTC can be seen in Figure 1.

## Competing interests

All authors declare they have no competing interests.

## Authors' contributions

ME participated in concept and software design, software writing, and manuscript writing; MW participated in software design and writing; KB participated in software design and writing; KH participated in software writing; AS participated in software design and writing; BO participated in software design; JK participated on concept design; PR conceived the concept and participated in concept design and manuscript writing. All authors read and approved the final manuscript.

## Declarations

We graciously acknowledge the resources provided for this work by grants from the NIH (R01AG042611), the Alzheimer's Association (MNIRG-11-205368), and startup funds from Brigham Young University.

This article has been published as part of *BMC Bioinformatics *Volume 15 Supplement 7, 2014: Selected articles from the 10th Annual Biotechnology and Bioinformatics Symposium (BIOT 2013). The full contents of the supplement are available online at http://www.biomedcentral.com/bmcbioinformatics/supplements/15/S7
